# Top 50 Cited Bone Graft Orthopedic Papers

**DOI:** 10.7759/cureus.23419

**Published:** 2022-03-23

**Authors:** Mohamed Elshohna, Nicholas Tsouklidis

**Affiliations:** 1 Orthopaedics, California Institute of Behavioral Neurosciences & Psychology, Fairfield, USA; 2 Health Care Administration, University of Cincinnati Health, Cincinnati, USA; 3 Medicine, California Institute of Behavioral Neurosciences & Psychology, Fairfield, USA; 4 Medicine, Atlantic University School of Medicine, Gros Islet, LCA

**Keywords:** impactful articles, bone grafts, bone tissue engineering, bone graft substitutes, top cited, bibliometric analysis

## Abstract

The purpose of this research is to recognize the highest 50 most-mentioned articles in the literature concentrating on bone grafts. That has been accomplished with the use of the Scopus database and the search slogan "bone grafts," and we inquired for the 50 most-cited articles on bone grafting. The study was completed in September 2020. We investigated the articles issued between 1970 and 2020. The articles were organized and classified based on the total number of citations. We appraised the following information relating to each article: first author, year of publication, journal, and title.

A total of 1,580 studies matched our search standards, of which the 50 most-cited extended between 1,862 and 403 citations. Seven articles were cited more than 1,000 times. The article by Marx et al. was the maximum-cited article, with 1,862 citations, followed by Younger et al.'s with 1,461 and Giannoudis et al.'s with 1,245. The majority of the studies originated from the United States (n = 30) and were published in the 2000s. Biomaterials was the most regular destination journal (n = 8), followed by the Journal of Bone and Joint Surgery American series (n = 7). A maximum of the articles focused on the different types of bone grafts and their alternatives including bone tissue engineering (n=29). Our investigation of the highest 50 articles linking to bone grafting has emphasized the most significant papers in the field. These cover a wide-ranging variety of topics including types, management, and mechanism of action of bone grafts. To recognize the present treatment guidelines and how the use of bone grafting has grown, it is vital to know the most-cited articles relating to this grafting.

## Introduction and background

The natural science of fracture healing is better recognized than ever before, with developments in orthopedic implants such as locked plates and bioabsorbable screws, and the osseous healing has become more expectable and less eventful. Nevertheless, occasionally one’s intrinsic biological response, or simultaneous surgical stabilization, is insufficient. With the hope of facilitating bone union, bone grafts, bone substitutes, and orthobiologics are being depended on more than ever before. The osteogenic, osteoconductive, and osteoinductive properties of these substrates have been illuminated in the basic science literature and authorized in the clinical orthopedic practice. Furthermore, business constructed around these substances is more fruitful and desirable than ever before. This analysis provides a wide-ranging overview of the basic science, clinical value, and economics of bone grafts, orthobiologics, and bone substitutes [1].

Within the academic medical field, the number of times an article is quoted by other writers has been commonly considered to be a dependable pointer of its academic influence and effect within this field [2]. Since Lefaivre et al. determined the 100 utmost-cited articles in the orthopedic field [3], there have been abundant reports categorizing the most-referenced articles across a wide range of orthopedic surgery subspecialties and subject ranges, including shoulder, hand, foot and ankle, arthroscopic surgery, hip arthroplasty, and trauma surgery [4-9].

The design of this research was to scrutinize the 50 most-cited articles in bone grafting in orthopedics and the features that make them significant to physicians and researchers within the orthopedic field. To achieve this goal, data from the Scopus citation indexing service were used to achieve an inclusive, organized citation search of all orthopedic-specific publications journal by journal. Given the nature of the field, we theorized that a noteworthy share of the detected citations would be basic science studies.

## Review

Method

The 50 most-cited articles linked to bone grafting were examined in the Scopus engine by using defined search terms. All forms of scientific papers, reviews, and conference papers with reference to our subject were graded along with the absolute number of citations and scrutinized for the following features: journal title, year of publication, number of citations, citation density, geographic origin, and article type. Mean citation number was considered as the total number of citations the article has established divided by the number of years since publication (total citations/years since publication) [10].

Results

The highest 50 articles concerning bone grafting have been cited a total of 33,895 times. The average number of citations per year is 753.22. The maximum 50 articles, numbers of citations, and mean citation number are listed in Table 1.

**Table 1:** Top 50 cited research papers relating to bone grafting.

	First author	Title	Citations	Citations /year
1	R.E. Marx [11]	Platelet-rich plasma: growth factor enhancement for bone grafts	1862	83
2	E.M. Younger [12]	Morbidity at bone graft donor sites	1461	47.13
3	P.V. Giannoudis [13]	Bone substitutes: an update	1245	83
4	A.J. Salgado [14]	Bone tissue engineering: state of the art and future trends	1120	70
5	S. Bose [15]	Recent advances in bone tissue engineering scaffolds	1115	139.38
6	E. Arrington [16]	Complications of iliac crest bone graft harvesting	1096	45.67
7	G. Ian Taylor [17]	The free vascularized bone graft: a clinical extension of microvascular techniques	1045	23.22
8	A.R. Amini [18]	Bone tissue engineering: recent advances and challenges	995	124.38
9	J.C. Banwart [19]	Iliac crest bone graft harvest donor site morbidity: a statistical evaluation	992	39.68
10	E. Carragee [20]	A critical review of recombinant human bone morphogenetic protein-2 trials in spinal surgery: emerging safety concerns and lessons learned	906	100.67
11	T.W. Bauer [21]	Bone graft materials: an overview of the basic science	833	41.65
12	C. Damien [22]	Bone graft and bone graft substitutes: a review of current technology and applications	812	28
13	H. Burchardt [23]	The biology of bone graft repair	755	20.41
14	M. Kikuchi [24]	Self-organization mechanism in a bone-like hydroxyapatite/collagen nanocomposite synthesized in vitro and its biological reaction in vivo	736	38.74
15	G.E. Friedlaender [25]	Osteogenic protein-1 (bone morphogenetic protein-7) in the treatment of tibial nonunions	733	38.58
16	R. Dimitriou [26]	Bone regeneration: current concepts and future directions	689	76.56
17	J.M. Kanczler [27]	Osteogenesis and angiogenesis: the potential for engineering bone	671	55.92
18	J. Goulet [28]	Autogenous iliac crest bone graft: complications and functional assessment	666	28.96
19	C.G. Finkemeier [29]	Bone-grafting and bone-graft substitutes	654	36.33
20	A.W. Yasko [30]	The healing of segmental bone defects, induced by recombinant human bone morphogenetic protein (rhBMP-2). A radiographic, histological, and biomechanical study in rats	641	22.89
21	J. Silber [31]	Donor-site morbidity after anterior iliac crest bone harvest for single-level anterior cervical discectomy and fusion	638	37.53
22	M. Yaszemski [32]	Evolution of bone transplantation: molecular, cellular, and tissue strategies to engineer human bone	606	25.25
23	H. Wang [33]	Biocompatibility and osteogenesis of biomimetic nano-hydroxyapatite/polyamide composite scaffolds for bone tissue engineering	600	46.15
24	P. Hernigou [34]	Percutaneous autologous bone-marrow grafting for nonunions: influence of the number and concentration of progenitor cells	597	39.8
25	T.J. Herbert [35]	Management of the fractured scaphoid using a new bone screw	589	16.36
26	L.T. Kurz [36]	Harvesting autogenous iliac bone grafts: a review of complications and techniques	587	18.94
27	R. Dimitriou [37]	Current concepts of molecular aspects of bone healing	578	38.53
28	S. Boden [38]	Use of recombinant human bone morphogenetic protein-2 to achieve posterolateral lumbar spine fusion in humans: a prospective, randomized clinical pilot trial 2002 Volvo award in clinical studies	554	30.78
29	R. Murugan [39]	Biomimetic nanocomposites for bone graft applications	552	36.8
30	J. Woodard [40]	The mechanical properties and osteoconductivity of hydroxyapatite bone scaffolds with multi-scale porosity recombinant human bone morphogenetic protein-2	542	41.69
31	P. Warnke [41]	Growth and transplantation of a custom vascularized bone graft in a man	525	32.81
32	M. Geiger [42]	Collagen sponges for bone regeneration with rhBMP-2	522	30.71
33	S. Laurie [43]	Donor-site morbidity after harvesting rib and iliac bone	515	14.31
34	H. Mankin [44]	Long-term results of allograft replacement in the management of bone tumors	506	21.08
35	W.R. Moore [45]	Synthetic bone graft substitutes	492	25.89
36	J. Zins [46]	Membranous versus endochondral bone: implications for craniofacial reconstruction	489	13.22
37	H. Frost [47]	A 2003 update of bone physiology and Wolff s law for clinicians	453	28.31
38	W. Bonfield [48]	Hydroxyapatite reinforced polyethylene - a mechanically compatible implant material for bone replacement	448	11.49
39	H. Yuan [49]	Osteoinductive ceramics as a synthetic alternative to autologous bone grafting	443	44.3
40	P. Francis [50]	Bone morphogenetic proteins and a signaling pathway that controls patterning in the developing chick limb	438	16.85
41	S. Khan [51]	The biology of bone grafting	435	29
42	D. Tadic [52]	A thorough physicochemical characterization of 14 calcium phosphate-based bone substitution materials in comparison to natural bone	435	27.19
43	E. Ahlmann [53]	Comparison of anterior and posterior iliac crest bone grafts in terms of harvest-site morbidity and functional outcomes	434	24.11
44	J. Inzana [54]	3D printing of composite calcium phosphate and collagen scaffolds for bone regeneration	433	72.17
45	W. De Long [55]	Bone grafts and bone graft substitutes in orthopedic trauma surgery: a critical analysis	423	32.54
46	O. Bergland [56]	Elimination of the residual alveolar cleft by secondary bone grafting and subsequent orthodontic treatment	421	12.38
47	P. Hernigou [57]	Treatment of osteonecrosis with autologous bone marrow grafting	416	23.11
48	G. Daculsi [58]	Biphasic calcium phosphate concept applied to the artificial bone, implant coating and injectable bone substitute	413	18.77
49	A. Oryan [59]	Bone regenerative medicine: classic options, novel strategies, and future directions	412	68.67
50	A. Greenwald [60]	Bone-graft substitutes: facts, fictions, and applications	408	21.4

The most commonly cited paper was by R.E. Marx et al. in 1998 representing a greater bone density in bone grafts with platelet-rich plasma with a total of 1,862 citations (mean citations 83/year) [11]. The most primitive publication was in 1975 by G. Ian Taylor et al. indicating a novel technique of free vascularized bone graft technique used and combined with a suitable soft tissue flap repairing method, where this system was established to salvage two injured legs which would otherwise have been amputated [17]. The newest publications were in 2014 by J. Inzana about a new category of bone graft technique which used low-temperature 3D printing of calcium phosphate scaffolds with greater functioning over old-style methods [54], and by A. Oryan who studied the literature of bone grafting and presented bone tissue engineering as an approach in the orthopedic surgery [59].

The maximum frequent decade in this list was the 2000s with 24 papers (Table 2).

**Table 2:** Top 50 papers published by decade.

Decade	Number
1970s	1
1980s	8
1990s	10
2000s	24
2010s	7

Twenty-six journals were included in publishing the maximum of 50 articles (Table 3). Impact factors of these journals fluctuated between 0.372 and 59.102. Journal of Biomaterials occupied the upper position of this list with eight publications (16%) and chased closely by the Journal of Bone and Joint Surgery - American Volume (n = 7) (14%) and Clinical Orthopaedics and Related Research (n = 6) (12%). The English language was the common language in all papers.

**Table 3:** Top 50 papers published per medical journal.

Medical journal	Number	Impact factor 2018
Biomaterials	8	10.273
Journal of Bone and Joint Surgery - Series A	7	4.716
Clinical Orthopaedics and Related Research	6	4.154
Spine	4	3.024
Plastic and Reconstructive Surgery	3	3.682
Injury	2	1.620
Angle Orthodontist	1	2.028
ANZ Journal of Surgery	1	1.071
Advanced Drug Delivery Reviews	1	16.663
BMC Medicine	1	8.639
Cleft Palate Journal	1	1.395
Composites Science and Technology	1	6.808
Critical Reviews in Biomedical Engineering	1	0.660
Development	1	5.763
European Cells and Materials	1	3.682
Journal of Applied Biomaterials: An Official Journal	1	0.372
Journal of Bone and Joint Surgery - Series B	1	4.301
Journal of Orthopaedic Surgery and Research	1	1.907
Journal of Orthopaedic Trauma	1	1.758
Lancet	1	59.102
Macromolecular Bioscience	1	2.895
Oral Surgery, Oral Medicine, Oral Pathology, Oral Radiology, and Endodontics	1	1.791
Proceedings of The National Academy of Sciences of The United States of America	1	9.553
Spine Journal	1	2.903
The Journal of the American Academy of Orthopaedic	1	2.441
Trends in Biotechnology	1	12.068
Total	50	

The highest 50 articles were created from 12 diverse countries (Table 4), where the USA was in the topmost with 30 articles (60%), then the UK with five articles (10%). Twenty-nine research papers are available as articles, while 15 reviews are involved in the uppermost cited papers and the conference papers are demonstrated six times (Table 5).

**Table 4:** Countries of top 50 research papers.

Country	Frequency	Percent
USA	30	60.0
UK	5	10.0
Australia	3	6.0
France	3	6.0
Germany	2	4.0
Iran	1	2.0
Japan	1	2.0
Netherlands	1	2.0
Norway	1	2.0
Portugal	1	2.0
Singapore	1	2.0
China	1	2.0
Total	50	100.0

**Table 5:** The origin of top 50 papers.

Origin	Frequency
Article	29
Conference paper	6
Review	15
Total	50

**Figure 1:**
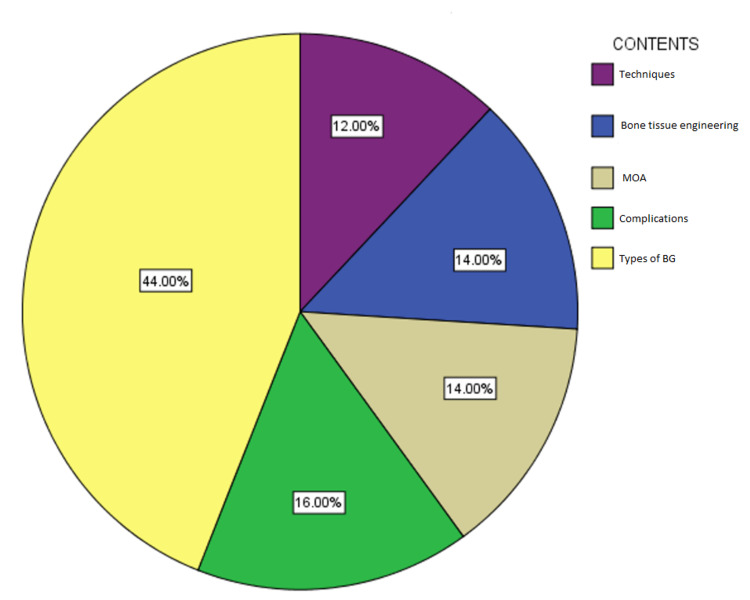
The contents of the top 50 papers. The contents are techniques, bone tissue engineering, mechanism of action (MOA), complications, and types of bone graft (BG).

A number of significant subjects are demonstrated in this list of top 50 papers. Twenty-nine articles (58%) scrutinize several categories of bone grafting. Besides these, seven papers are focused on bone tissue engineering, which points to inducing a novel practical bone regeneration method through a synergetic combination of biomaterials, cells, and numerous growth factors. Eight papers (16%) observe bone grafting complications that are frequently connected to the iliac bone graft donor site. The mechanism of action of the bone graft method in the acceleration of bone healing is clearly demonstrated in seven papers (14%); additionally, the same numbers of papers (seven) are focused on proving various techniques in applying bone grafts (Figure 1).

Discussion

Our study recognizes the topmost 50 research papers published on bone graft based on the number of citations recognized in several scientific studies. This research validates a wide range of valued information regarding the authors, topics, and time periods that have had a deep impact on the orthopedic specialty. It records the changes in information over 45 years. In this paper, the citation number was nominated as the marker of effect. This has been carried out for several further surgical specialties including numerous orthopedic topics. Citation analysis, although controversial, allows for the measurement of peer recognition and suggests insights into the readership of the article [61]. Regrettably, the citation number does not directly associate with study quality. Nevertheless, a high citation number specifies that various researchers have found an article beneficial and its material worthy for inclusion and more discussion in their work.

The 50 uppermost cited articles on bone graft were cited 33,895 times. The highest seven papers, which were cited more than 1,000 times, according to absolute numbers were cited at nearly 9,000 times. These numbers are higher than the uppermost cited papers in the numerous orthopedic fields such as hip and knee arthroplasty and oncology [62,63]. This is even more obvious, when compared to the uppermost cited papers in hand or shoulder surgery [4,5].

The most-cited paper illustrated the mechanism of action of platelet-rich plasma in improving the usefulness of bone grafts by creating a higher concentration of human platelets and platelet-derived growth factors (1998) issued in the Oral Surgery, Oral Medicine, Oral Pathology, Oral Radiology, and Endodontics [11]. This study has been cited 1,862 times with a mean citation number of 38.00/year. In this paper, Marx reached an assumption that the addition of platelet-rich plasma to various bone grafts augmented the radiographic maturation rate 1.62 to 2.16 times when compared to bone grafting without platelet-rich plasma [11].

The second maximum-cited paper was by Younger Edward M (1989) about complications at bone graft donor sites published in the Journal of Orthopaedic Trauma. This research was cited 1,461 times (47.13 citations/year). Younger studied the medical records of 239 patients with 243 autogenous bone grafts taken on to document the morbidity at the donor sites. He stated that the general major complications were deep infection, prolonged wound drain, hematomas collection, reoperation, pain lasting for more than six months, severe sensory loss, and unsightly scars, while the minor complications comprised superficial infection, minor wound problems, temporary sensory loss, and mild or resolving pain. He observed that there was a much higher complication rate if the incision used for the surgery was also the same incision used to harvest the bone graft [12].

A whole of 12 countries contributed to the uppermost 50 articles with the majority derived from the USA. Forty-four papers were created from countries where English is the first language. All countries characterized on the list are first-world countries with a large health-care expenditure [64]. Parallel results have been realized in other fields where the USA led most positions [3,65,66].

Remarkably, the uppermost five articles were published in a 23-year gap from 1989 to 2012. Consequently, they have had significant time to merge these top citation numbers and this appears to be a crucial factor in their top positions. When we investigate the mean citation number of the topmost two articles, their citation densities are obviously high at 83 and 47.13 correspondingly. Though, the uppermost citation density is noticed in the fifth paper (Recent advances in bone tissue engineering scaffolds) at 139.38 citations/year [15]. This recommends that these papers are highly significant in the field. Nevertheless, a limitation in mean citation number does not signify the progression of a paper's influence over time. For example, a paper that was published three decades ago about the free vascularized bone graft: A clinical extension of microvascular techniques by Professor Geoffrey Ian Taylor who was particularly recognized for his pioneering research in microsurgery and bone grafting and received extensive acknowledgment and frequent citations at that time may still hold a high mean citation number despite not being referenced for many years [17]. O’Neill (2014) recommended that the mean citation number may in fact be effective in evaluating the proximity of impact a paper has, when comparing articles from diverse time periods [67].

There are an additional number of boundaries related to this type of research documented by various authors. The Scopus search engine used in this work extends from 1996 to the present day. Hence, any articles published before this date will not be involved in our study, which likely results in numerous classic research articles being excluded. Citation analysis also brings with it some intrinsic faults. It does not take account of biased citing, self-citation, formal or informal influences not cited, technical limitations of citation indices, and not being able to add publications if not indexed in Scopus [68]. Alternative metrics, or Altmetrics, assess the influence of scholarly materials via online metrics, with an emphasis on data arising from social media outlets, for instance: mentions, views, shares, download, saves, tweeting, tags, and comments. Altmetrics will certainly provide a complimentary measurement through the internet to traditional citation metrics, which will certainly become an alternative dimension whereby the reach of a journal article can be evaluated [69].

## Conclusions

The scrutiny of the uppermost 50 articles connecting to bone grafting has emphasized the most significant papers in the field. These cover a wide range of issues including categories, management, and mechanism of action of bone grafting. Citation number was used to detect the influence of these papers. Although this may not directly associate with study quality, it does provide an insight into the effect that a research paper has had on the scientific community. This list may prove priceless to surgeons involved in the treatment of patients who need to use bone grafting in orthopedic surgeries, especially in replacing bone defects and motivating fracture healing and those actively advancing the progress of the field.
